# Ferroptosis: a promising candidate for exosome-mediated regulation in different diseases

**DOI:** 10.1186/s12964-023-01369-w

**Published:** 2024-01-02

**Authors:** Limin Liu, Yulin Ye, Rui Lin, Tianyu Liu, Sinan Wang, Zelin Feng, Xiaoli Wang, Hailong Cao, Xin Chen, Junming Miao, Yifei Liu, Kui Jiang, Zhibo Han, Zongjin Li, Xiaocang Cao

**Affiliations:** 1 Department of Gastroenterology and Hepatology, Tianjin Medical University General Hospital, Tianjin Medical University, Tianjin, 300052 China; 2 National Engineering Research Center of Cell Products, AmCellGene Engineering Co., Ltd, Tianjin, 300457 China; 3 Tianjin Key Laboratory of Engineering Technologies for Cell Pharmaceutical, Tianjin, 300457 China; 4 State Key Laboratory of Experimental Hematology, Institute of Hematology & Blood Diseases Hospital, Chinese Academy of Medical Sciences & Peking Union Medical College, 288 Nanjing Road, Tianjin, 300020 China; 5 Nankai University School of Medicine, Tianjin, 300071 China

**Keywords:** Exosomes, Stem cells, Ferroptosis, Therapeutic target, Signalling pathway, Iron metabolism, Lipid metabolism

## Abstract

Ferroptosis is a newly discovered form of cell death that is featured in a wide range of diseases. Exosome therapy is a promising therapeutic option that has attracted much attention due to its low immunogenicity, low toxicity, and ability to penetrate biological barriers. In addition, emerging evidence indicates that exosomes possess the ability to modulate the progression of diverse diseases by regulating ferroptosis in damaged cells. Hence, the mechanism by which cell-derived and noncellular-derived exosomes target ferroptosis in different diseases through the system Xc^−^/GSH/GPX4 axis, NAD(P)H/FSP1/CoQ10 axis, iron metabolism pathway and lipid metabolism pathway associated with ferroptosis, as well as its applications in liver disease, neurological diseases, lung injury, heart injury, cancer and other diseases, are summarized here. Additionally, the role of exosome-regulated ferroptosis as an emerging repair mechanism for damaged tissues and cells is also discussed, and this is expected to be a promising treatment direction for various diseases in the future.

Video Abstract

**Supplementary Information:**

The online version contains supplementary material available at 10.1186/s12964-023-01369-w.

## Background

Ferroptosis is an iron-dependent form of programmed cell death that was defined in 2012. It is characterized by the excessive accumulation of lipid peroxides and reactive oxygen species (ROS) in cells and is distinct from apoptosis, necrosis, and autophagy [1, 2]. As an evolutionarily conserved process, ferroptosis plays a vital role in the developmental processes of diverse organisms, including both animals and plants [3, 4]. Recent studies on neurological diseases have indicated that the presence of iron metabolism imbalance is closely linked to several pathological characteristics of Alzheimer's disease and that excessive iron accumulation within the brain has been identified as a potential cause for the accelerated cognitive deterioration observed in individuals affected by Alzheimer's disease [5]. Regarding cardiovascular diseases, myocardial apoptosis is an important pathological change in heart failure. Furthermore, research has demonstrated that in addition to apoptosis, ferroptosis is also involved in the progression of heart failure [6, 7]. This phenomenon is also found in liver disease. In the course of nonalcoholic steatotic liver disease, ferroptosis may be a predisposing factor of the inflammatory response; moreover, ferroptosis in hepatocytes may precede apoptosis in the early stage of inflammation [8]. Ferroptosis of human bronchial epithelial cells was also observed in an LPS-induced acute lung injury model [9]. Furthermore, ferroptosis has been found to be involved in the pathological process of acute kidney injury mediated by ischaemia–reperfusion injury (IRI) after cardiac surgery due to the iron released from cardiopulmonary bypass. If this condition is not controlled in time, then increases in the cellular labile iron pool (LIP) can occur [10]. In orthopaedic diseases, it has also been confirmed that iron overload is positively correlated with the occurrence and development of osteoporosis and then inhibits the activity of osteoblasts [11]. In addition, the occurrence and development pathways of some cancers are closely related to ferroptosis, and the survival and proliferation of tumour cells are easily affected by ferroptosis [12]. Given that ferroptosis is closely related to the occurrence and development of several diseases, it is essential to develop new strategies against ferroptosis.

Cellular therapy, a novel therapeutic strategy for multiple diseases, has been a research hotspot in recent years. However, cell transplantation therapy still has many limitations, which impedes its application in the clinic [13]. Currently, there is abundant evidence showing that mesenchymal stem cells exert their biological effects by secreting paracrine mediators, especially exosomes [14]. As an important carrier of intercellular communication, exosomes containing a variety of proteins, RNA, and other components play a crucial role in disease diagnosis and treatment by regulating the inflammatory response, angiogenesis and cell protection after injury [15, 16]. Notably, exosomes have been shown to participate in disease progression by regulating ferroptosis [17]. Since the association between exosomes and ferroptosis has been noted, an increasing number of researchers have conducted intensive studies in this field.

This review will focus on the main mechanisms by which exosomes from different sources regulate ferroptosis in different diseases. Subsequently, the application of ferroptosis as an exosome regulatory target in related diseases will be introduced. It is worth noting that exosome-regulated ferroptosis is a "double-edged sword", which suggests the importance for regulating disease progression by reasonable induction or inhibition of ferroptosis (Tables 1 and 2). Additionally, the direction with the most potential for diagnosing and treating different diseases is also provided.

**Table 1:** Mechanism by which exosomes enhance ferroptosis in different diseases

System	Disease types	Exosomes sources	Molecular mediators	Target	Biological effects	References
Digestive system	Liver fibrosis	HucMSC	BECN1	xCT/GPX4	Promote ferroptosis of HSC and inhibit liver fibrosis	[18]
Cardiovascular system	Obesity-induced myocardial injury	ATM	miR-140-5p	SLC7A11/GSH	Promote ferroptosis of cardiac myocyte and aggravate heart injury	[19]
	Atrial fibrillation	CF	miR-23a-3p	SLC7A11	Promote ferroptosis of cardiac myocyte and worsen disease	[20]
Nervous system	sepsis associated encephalopathy	serum	NEAT1	miR-9-5p/TFRC and GOT1	Promote ferroptosis of brain microvascular endothelial cells and lead to SAE	[21]

*HucMSC* human umbilical cord mesenchymal stem cell-derived exosomes, *ATM* adipose tissue macrophage, *CF* cardiac fibroblast, *GOT1* glutamic oxaloacetic transaminase 1

**Table 2:** Mechanism of exosomes against ferroptosis in different diseases

System	Disease types	Exosomes sources	Molecular mediators	Target	Biological effects	References
Digestive system	Acute liver injury	MSC	CD44/OTUB1	SLC7A11/system Xc-	Inhibit ferroptosis of hepatocyte and alleviate acute liver injury	[22]
	Liver fibrosis	HBV-LO2 cells	miR-222	TFRC	Inhibit the ferroptosis of LX2 cells and promote liver fibrosis and liver failure	[23]
	Steatotic liver IRI	HO-1/BMMSCs	miR-29a-3p	IREB2	Inhibit ferroptosis of steatotic liver IRI and improve liver tissue and cell injury	[24]
			miR-124-3p	STEAP3		[25]
	Gastric carcinoma	CAFs	miR-522	ALOX15	Inhibit ferroptosis of gastric cancer cells and reduce chemotherapy sensitivity	[26]
		GC	lncFERO	hnRNPA1/SCD1	Inhibit ferroptosis of GCSC and enhance the chemotherapy resistance of GCSCs	[27]
	Colorectal carcinoma	Adipose cell	MTTP	PRAP1/ZEB1、GPX4/xCT	Inhibit ROS production and ferroptosis in CRC cells and promote tumor growth	[28]
	Pancreatic carcinoma	PANC	GOT1	Nrf2/HO-1	Inhibit ferroptosis of cancer cells and promote the development of tumors	[29]
Cardiovascular system	Acute myocardial infarction	HucMSC	miR-23a-3p	DMT1	Inhibit ferroptosis of cardiac myocyte and reduce myocardial injury	[30]
		Pericardial adipose tissues	Adipsin	IRP2	Inhibit cardiomyocyte ferroptosis and maintain iron homeostasis	[31]
Nervous system	Cerebral hemorrhage	ADSC	miR-19b-3p	IRP2	Inhibit the ferroptosis of damaged brain cells and relieve symptoms	[32]
	Acute spinal cord injury	MSC	lncGm36569	miR-5627-5p/FSP1	Inhibit ferroptosis of neuronal cells and attenuate the neurological dysfunction caused by injury	[33]
	Cerebral IRI	BMMSC-EVs/Fer-1	Fer-1	GPX4/COX2	Inhibit ferroptosis of neurons and alleviate brain injury in mice	[34]
Respiratory system	Non-small cell lung cancer	NSCLC-cells	miR-4443	METTL3/FSP1	Inhibit ferroptosis of tumor cells and promote cisplatin resistance in NSCLC	[35]
	Lung adenocarcinoma	Plasma	cir93	AA/ACSL4、LPCAT3	Inhibit ferroptosis in lung adenocarcinoma cells and decrease lipid peroxidation in cancer cells	[36]
	Osteoporosis	EC	—	Ferritin	Inhibit ferroptosis of osteoblast and reverse the glucocorticoid-induced osteoporosis	[37]
Urinary System	Acute kidney injury	USC	lncTUG1	SRSF1/ACSL4	Inhibit ferroptosis of renal tubular epithelial cells and alleviate acute kidney injury	[38]
Others	UVB-induced skin injury	ADSC	circ-Ash1l	miR-700-5p/GPX4	Inhibit the ferroptosis of cells and improve UVB-induced skin damage	[39]
	Diabetic foot ulcer	BMMSC	circ-ITCH	TAF15/Nrf2	Inhibit ferroptosis of venous endothelial cells and promote angiogenesis and wound healing	[40]

*MSC* mesenchymal stem cell, *HBV-LO2 cells* HBV-infected LO-2 cells, *HO-1/BMMSCs* bone marrow mesenchymal stem cells by heme ox ygenase-1, *CAFs* cancer-associated fibroblasts, *GC* gastric cancer, *PANC* pancreatic cancer, *ADSC* adipose-derived stem cells, *BMMSC-EVs/Fer-1* Fer-1 loaded bone marrow mesenchymal stem cell-derived extracellular vesicles, *NSCLC* non-small cell lung carcinoma, *EC* vascular endothelial cell, *USC* human urine-derived stem cells, *OTUB1* deubiquitination, *MTTP* microsomal triglyceride transfer protein, *GOT1* glutamic oxaloacetic transaminase 1, *SCD1* stearoyl-Coa-desaturase, *SRSF1* serine/arginine splicing factor 1, *TAF15* TATA-Box binding protein associated factor 15, *lncTUG1* lncRNA taurine-upregulated gene 1, *circ-ITCH* circRNA-itchy E3 ubiquitin protein ligase, *cir93* circRNA_101093, *hnRNPA1* heterogeneous nuclear ribonucleoprotein A1

## Overview of exosomes

Exosomes are derived from intracellular multivesicular bodies (MVBs) containing intraluminal vesicles, which is a category of extracellular vesicles with a size range of 40 to 160 nm (average ~ 100 nm) in diameter [41]. Exosomes carry biologically active molecules such as proteins, nucleic acids (mRNAs, microRNAs (miRNAs), long noncoding RNAs (lncRNAs), DNA, etc.), lipids, and other components [42]. Exosome-containing RNAs are the main substances regulating cell–cell communication. They can also regulate gene expression in recipient cells through their own degradation and re-expression and then participate in cell cycle progression, cell migration, angiogenesis, or histone modification [43–45]. In addition to RNAs, several exosomal proteins and lipids have also been reported. The composition of exosome lipids and proteins may affect their pharmacokinetic properties, and their natural components may play a part in enhancing bioavailability and reducing adverse reactions [41]. There are several surface markers on the exosomal membrane, such as transmembrane proteins (CD63, CD9, CD81), heat shock proteins, TSG101, and Alix, which can regulate processes such as fusion, migration, and adhesion [46–49]. Recent studies have shown that these exosomes can be secreted by different kinds of cells, including mesenchymal stem cells, immune cells, endothelial and epithelial cells, and various cancer cells [50–52]. In addition, noncell-derived exosomes, such as those in body fluids (blood, saliva, etc.) and food-derived exosomes, have also attracted extensive attention [53–56]. Because its composition, including its surface proteins and lipids, plays a vital role in its function, the selection of exosome sources is critical for treatment [57].

## Mechanism and application of exosome-regulated ferroptosis

Ferroptosis is a newly discovered mode of programmed cell death that exists in the pathological processes of various diseases. Therefore, it offers new insights into the development of new cell protection strategies and provides new targets for disease treatment. For example, cancer cells are highly sensitive to ferroptosis [58]. Thus, this sensitivity could be used to induce cancer cell ferroptosis and then eliminate cancer. Ferroptosis is also a new therapeutic target for cardiac diseases [59] and is also being investigated in the treatment of other organs and tissues as well as injuries. Here, the application of ferroptosis as a regulatory point for exosomes in the occurrence and development of different diseases has been presented, and the mechanisms of action and relationship between ferroptosis and exosomes have been revealed.

### Exosome-regulated ferroptosis by system Xc-/GSH/GPX4 and its application in treating diseases

#### The role of system Xc-/GSH/GPX4 in ferroptosis

System Xc- is an amino acid reverse transporter located on the cell membrane; it is mainly composed of the solute carrier family 3 member 2 (SLC3A2) and solute carrier family 7 member 11 (SLC7A11) [60]. System Xc- exchanges extracellular cystine with intracellular glutamic acid in a 1:1 ratio to maintain the balance of REDOX [61]. The ingested cystine undergoes an enzymatic reaction in the cell to form cysteine, which then participates in the biosynthesis of glutathione (GSH), a key factor in regulating ferroptosis. Therefore, the inhibitory effect on the activity of system Xc- affects the synthesis of GSH by inhibiting cystine absorption, which leads to the accumulation of peroxide in vivo that can induce ferroptosis [62]. Studies have shown that regulating the expression of the functional subunit SLC7A11 of system Xc- can affect the activity of system Xc-  [63]. The expression of SLC7A11 is regulated by several factors. For example, p53, nuclear factor, erythroid 2-like 2 (NFE2L2), BECN1, BRCA1-associated protein 1 (BAP1), and others can regulate SLC7A11 activity or expression [64–67].

The cysteine-GSH-GPX4 axis is a downstream node of system Xc- and has been considered a major factor in resistance to ferroptosis for many years [68, 69]. Glutathione peroxidase 4 (GPX4) is a key regulator of ferroptosis that inhibits ferroptosis by lowering hydroperoxides and blocking lipoxygenase pathways [70]. GPX4 is essential to preserve tissue homeostasis and avoid cell death during tissue injury [71]. Studies have shown that inactivation of GPX4 with RSL3 (an inducer of ferroptosis) and ML162 (an inhibitor of GPX4) can increase the production of lipid ROS, thus inducing ferroptosis [72, 73]. GPX4 activity and expression are not only regulated by GSH but also controlled by the amino acid selenocysteine in its active centre, which can be embedded into GPX4 through the transporter selenocysteine tRNA to maintain the activity of GPX4 and scavenge lipid ROS, thus inhibiting ferroptosis [4]. Therefore, blocking the GSH synthesis pathway or selenium deficiency leads to suppression of GPX4 activity and induces ferroptosis.

#### The application of exosome-regulated ferroptosis in treating different diseases through system Xc-/GSH/GPX4

Exosomes play different regulatory roles depending on the cell from which they are derived and the microenvironment in which they reside. Thus, in some diseases, exosomes of their own origin can regulate the ferroptosis of target cells via system Xc-/GSH/GPX4 to promote disease progression. Studies have shown that long-term consumption of a high-fat diet (HFD) causes myocardial damage through myocardial cell ferroptosis [74]. A HFD causes a sustained, low-grade inflammatory response in adipose tissue, which is maintained by inflammatory macrophages [75]. MiR-140-5p has been found to be highly expressed in adipose tissue macrophage-derived exosomes (ATM-Exo) in obesity-induced myocardial injury. Exosomal miR-140-5p targeting SLC7A11 inhibits GSH expression and promotes ferroptosis in cardiomyocytes, exacerbating damage to cardiomyocytes [19]. Similarly, in another myocardial injury model of atrial fibrillation, cardiac fibroblast-derived exosomes (CF-Exo) inhibit SLC7A11 expression and promote cardiac ferroptosis by delivering rich miR-23a-3p [20]. However, it also provides potential therapeutic targets for the treatment of obesity-related cardiomyopathy and for the continued development of atrial fibrillation.

Exogenous administration of mesenchymal stem cell-derived exosomes (MSC-Exos) to regulate ferroptosis in some diseases has also been widely studied. In an acute liver injury (ALI) model induced by carbon tetrachloride (CCL4), Lin et al. found that there was a high concentration of lipid peroxide accumulation in the liver. This accumulation caused the ubiquitination of SLC7A11 and its abnormal expression, leading to ferroptosis in liver cells. However, MSC-Exos can maintain the stability and function of SLC7A11 by increasing the expression of CD44 (regulating SLC7A11) and OTUB1 (deubiquitination), thus activating system Xc − to rescue ALI caused by ferroptosis in hepatocytes [22]. Cerebral ischaemia/reperfusion injury (I/R) causes irreversible damage to the brain and neurons [76]. Therefore, effectively preventing the occurrence of cerebral I/R is an issue that urgently needs to be addressed. By establishing cerebral I/R models in vivo and in vitro, Liu et al. found that Fer-1-loaded bone marrow mesenchymal stem cell-derived extracellular vesicles (BMMSC-EVs/Fer-1) could successfully deliver Fer-1 to neurons, reduce the occurrence of neuronal ferroptosis and inflammation, and alleviate cerebral I/R injury in mice. Further investigation of its mechanism showed that BMMSCs-EVs/Fer-1 could regulate the GPX4/COX2 axis and play a protective role in the brain by upregulating GPX4 and inhibiting COX2 expression [34]. In addition, exosome-regulated ferroptosis also plays an important role in skin injury and wound healing. Zha et al. found that hypoxia-pretreated adipose-derived stem cell (ADSC) exosomes (HExo) could reverse ROS generation by reducing miR-700-5p and increasing GPX4 expression via delivery of circ-Ash1l, thereby improving UV-induced skin damage [39]. Previous studies have shown that exosomes can promote wound healing in diabetic foot ulcers (DFUs), thereby alleviating their symptoms [77]. A recent study found that ferroptosis may play a significant role in this process. The circRNA-itchy E3 ubiquitin protein ligase (circ-ITCH) derived from BMMSC-Exo can recruit TATA-Box binding protein associated factor 15 (TAF15) to activate the Nrf2 signalling pathway, thereby inhibiting ferroptosis of venous endothelial cells, promoting angiogenesis, and accelerating the wound healing process of DFU mice [40].

While cellular ferroptosis may seem to be an unfavourable option, promoting ferroptosis may be beneficial for certain diseases. Increased activated hepatic stellate cells (HSCs) are a key point in the progression of liver fibrosis, and HSCs are considered to be an important target for the treatment of liver fibrosis. Tan et al. found that human umbilical cord mesenchymal stem cell-derived exosomes (HucMSC-Exo) could reduce the expression of xCT/GPX4 by transferring BECN1, thereby inducing ferroptosis and inhibiting the activation of HSCs, providing a new perspective for liver fibrosis treatment [18, 78]. In addition, Guo et al. found that GOT1 protein enrichment in exosomes secreted by pancreatic cancer cells inhibits ferroptosis of cancer cells and promotes tumour development. In terms of mechanism, GOT1 can upregulate the expression of the C–C motif chemokine receptor-2 (CCR2), thereby activating the Nrf2/HO-1 axis to resist ferroptosis in cancer cells [79].

### Exosome-regulated ferroptosis by the NAD(P)H/FSP1/CoQ10 system and its application in treating diseases

#### The role of the NAD(P)H/FSP1/CoQ10 system in ferroptosis

Ferroptosis inhibitor protein 1 (FSP1) is a key protein that protects against ferroptosis. FSP1 knockout cell lines are more sensitive to ferroptosis inducers and can be rescued by overexpression of FSP1 [29, 80]. As a member of the quinone oxidoreductase (NDH-2) family, FSP1 can reduce CoQ10 and reduce ubiquinone (CoQ) to dihydroubiquinone (CoQH2) in the plasma membrane and thereby prevent the accumulation of lipid peroxides. Moreover, Doll et al. found that FSP1 could catalyse the regeneration of COQ10 through NAD(P)H. In addition, FSP1 exerts a protective effect against ferroptosis induced by GPX4 deficiency. Collectively, the NAD(P)H/FSP1/CoQ10 pathway is another antioxidant pathway parallel to the classic glutathione-based GPX4 that works together with GPX4 to inhibit lipid peroxidation and ferroptosis [80, 81].

#### The application of exosome-regulated ferroptosis in treating different diseases through the NAD(P)H/FSP1/CoQ10 system

Exosomes in the NAD(P)H/FSP1/CoQ10 system mainly regulate the expression of FSP1 through their RNAs to affect ferroptosis of target cells and then participate in disease development. Exosomal miR-4443 has been reported to be a key regulator of oncogenesis and metastasis and a key mediator of drug resistance in tumours [82, 83]. Song et al. found that exosomal miR-4443 was enriched in clinical non-small cell lung carcinoma (NSCLC) resistant to cisplatin and exosomes of cisplatin-resistant NSCLC cell lines compared to cisplatin-sensitive tumour tissues. Through further bioinformatics analysis and luciferase assays, miR-4443 was shown to regulate FSP1 expression in an N6-methyladenosine-dependent manner through its target gene methyltransferase-like 3 (METTL3) and increase the resistance of tumour cells to ferroptosis, promoting resistance to cisplatin in NSCLC [84]. In addition, altered expression of ferroptosis-related markers was observed in an in vivo acute spinal cord injury (ASCI) model [35]. Shao et al. established in vitro models of ASCI mice and hypoxic cell in vivo models and found that MSC-Exos were able to inhibit ROS production by upregulating FSP1 expression. Therefore, damaged neurons are protected from ferroptosis, and the proliferation of hypoxic nerve cells and neurological dysfunction are restored in ASCI mice. Further mechanistic study found that LncGm36569, which was highly expressed in MSC-Exos, could bind to its competing endogenous RNA, miR-5627-5p, to upregulate the expression of its target gene FSP1, thereby inhibiting ferroptosis and promoting the repair of nerve function [85–87].

### Exosome-regulated ferroptosis by the iron metabolism pathway and its application in treating diseases

#### The role of the iron metabolism in ferroptosis

Iron is one of the essential trace elements in the body and is essential for cell survival. However, iron metabolism disorders, especially iron overload, promote ROS generation and accumulation through the Fenton reaction, thus triggering ferroptosis [33, 88]. Extracellular iron (Fe^3+^) can form a complex with circulating transferrin (TF) and bind to a specific membrane transferrin receptor protein-1 (TFR1) in cell endosomes. Subsequently, Fe ^3+^ in the nucleus is reduced to ferrous iron (Fe ^2+^) by six-transmembrane epithelial antigen of prostate 3 metalloreductase (STEAP3). Then, mediated by divalent metal transporter 1 (DMT1) or zentz-iron regulatory protein family 8/14 (Zip8/14), Fe ^2+^ is released into the cytoplasm and stored in LIP or ferritin [89]. Ferritin is the main intracellular iron storage protein complex, consisting of two subunits, ferritin light peptide 1 (FTL1) and ferritin heavy peptide 1 (FTH1) [90]. Moreover, Fe ^2+^ can also be transported out of cells via ferroprotein (FPN) [91]. Ferritin can be degraded by autophagy and is regulated by nuclear receptor coactivator 4 (NCOA4). Thus, a large amount of free Fe ^2+^ is released, which increases the accumulation of intracellular iron and ROS and leads to cell ferroptosis [92]. In addition, iron response element binding protein 2 (IREB2), a major regulator of iron metabolism, regulates the expression of TRFC, FTH1, and FTL [93]. Fe ^2+^ entering cells can transport iron out of cells through DMT1, ferritin MVBs, and exosomes. Inhibition of DMT1 or blocking of MVBs can limit intracellular iron outflow and thus increase the intracellular iron level and induce cell ferroptosis. Notably, Brown et al. demonstrated that extracellular vesicles, especially exosomes, could expel intracellular iron when stimulated by ferroptosis, which is an important mechanism driving ferroptosis resistance [94, 95]. In short, increased iron intake, decreased iron storage, or decreased iron outflow can lead to iron overload, which can lead to ferroptosis.

#### The application of exosome-regulated ferroptosis in treating different diseases through the iron metabolism pathway

It is known that the maintenance of intracellular iron homeostasis is very important for cell survival and even for normal functioning of the body. As a result, in some diseases, self-derived exosomes can regulate the progression of ferroptosis by regulating proteins involved in iron metabolism, leading to an exacerbation of the disease. Sepsis induces sepsis-associated encephalopathy (SAE) and aggravates the progression of the disease. Through a rat model of sepsis, Wei et al. found that the blood–brain barrier of septic rats was damaged and that the levels of the serum exosome nuclear-enriched transcript 1 (NEAT1), a type of lncRNA, were significantly increased [21, 96]. Previous studies have shown that NEAT1 acts as a ceRNA of miR-9-5p, and its target genes are TFRC and glutamic oxaloacetic transaminase 1 (GOT1), an iron export protein that is essential for iron transport between different cells [97, 98]. NEAT1 sponging miR-9-5p in exosomes promotes the expression of TFRC and GOT1, induces ferroptosis of brain microvascular endothelial cells, leads to SAE and aggravates disease [21]. Similarly, exosomal miR-222 from HBV-infected LO-2 cells inhibits ferroptosis by inhibiting TFRC and promotes the activation of the hepatic stellate cell line LX-2. This leads to liver fibrosis and ultimately accelerates the development of liver failure [23]. In contrast, as an important adipokine, the effect of adipsin on injured cardiomyocytes after myocardial infarction (MI) is surprising. Man et al. found that exosomes derived from pericardial adipose tissue can effectively deliver adipsin to myocardial tissue. They can also interact with iron regulatory protein 2 (IRP2) to upregulate the level of FTH in the peri-infarct area and downregulate the level of transferrin TFR to maintain iron homeostasis, thereby protecting cardiomyocytes against ferroptosis [99, 100].

BMMSC transplantation is an important option for the treatment of a variety of diseases, which can enter damaged tissues and contribute to tissue repair [31]. However, interference with the internal environment and metabolism can affect efficacy, especially since the latter is prone to ferroptosis [101, 102]. Studies have found that modification of BMMSCs (HO-1/BMMSCs) by haem oxygenase-1 (HO-1), an antioxidant enzyme that protects cells from oxidative stress, can prolong their retention time in vivo and optimize their paracrine function, thereby enhancing the enrichment and function of exosomes [103, 104]. Hepatic IRI is an inevitable outcome after a liver transplant. However, steatotic liver, a common type of enlarged standard donor liver, is sensitive to postoperative IRI and poorly tolerated [105, 106]. Hepatic IRI is also closely related to the abnormal expression of iron metabolism-related proteins leading to ferroptosis [107]. Therefore, Li and his team constructed the IRI model of fatty liver and the HR model of fatty liver cells (SHP-HR). They found that the expression of IREB2 was increased in steatotic liver IRI and SHP-HR, which led to ferroptosis of hepatocytes and further aggravated liver tissue damage. HO-1/BMMSC-derived exosomes enriched with miR-29a-3p, which downregulates IREB2, ameliorated liver tissue and hepatocyte injury and inhibited ferroptosis in vitro and in vivo [108]. In the same year, the team used the rat liver transplantation model with severe steatotic donor liver as the donor and the SHP-HR model to verify again that HO-1/BMMSC-derived exosomes were involved in the regulation of hepatocyte ferroptosis. To further investigate its intrinsic mechanism, Song et al. used miRNA sequencing technology to screen upregulated miRNAs in exosomes and analysed the target genes of the upregulated miRNAs. They found that STEAP3 could interact with miR-124-3p. HO-1/BMMSC-derived exosomes enriched with miR-124-3p can reduce the degree of ferroptosis of hepatocytes by inhibiting STEAP3, thereby alleviating liver injury [24]. In conclusion, these studies provide a new therapeutic target for IRI in steatosis donor livers and a new exosome transplantation strategy for exosome-regulated ferroptosis.

In addition to HO-1/BMMSCs, Yi et al. experimentally demonstrated that ADSCs overexpressing miR-19b-3p could efficiently package it into their exosomes. Exosomes can deliver miR-19b-3p to the site of intracerebral haemorrhage to inhibit ferroptosis and reduce cell damage. It has been shown that miR-19b-3p achieves these results by targeting IRP2 [25]. In addition, exosomes themselves are rich in miRNAs. The transplantation of HucMSC-derived exosomes enriched with miR-23a-3p has been shown to improve myocardial infarction. It is worth noting that the expression of DMT1 was significantly higher in mice with acute myocardial infarction at 24 and 48 h than in mice in the control group. Further studies showed that miR-23a-3p could reduce myocardial injury by targeting DMT1 to improve cardiac myocyte ferroptosis [32]. Furthermore, vascular endothelial cell-derived exosomes (EC-Exos) have been identified as promising targeted nanoparticles, especially in the skeletal system [30]. Using Kyoto Encyclopedia of Genes and Gnomes (KEGG) pathway enrichment analysis, Yang et al. found that dexamethasone treatment induced activation of the ferroptosis pathway. EC-Exos could resist glucocorticoid-induced inhibition of osteoblasts by inhibiting ferrophagy-dependent ferroptosis in vitro and in vivo, thereby alleviating osteoporosis [109].

### Exosome-regulated ferroptosis by the lipid metabolism pathway and its application in treating diseases

#### The role of the lipid metabolism in ferroptosis

The heart of ferroptosis is the accumulation of iron-dependent lipid peroxides, so lipid metabolism is critical to the occurrence and development of ferroptosis. It has been shown that ROS can react with polyunsaturated fatty acids (PUFAs) on lipid membranes to cause lipid peroxidation, which is one of the necessary stages for ferroptosis to eventually occur. Free PUFAs are substrates for the synthesis of lipid signal transduction mediators. They are esterified by acyl-CoA synthase long chain family member 4 (ACSL4) and incorporated into membrane phospholipids (PLs) with the help of recombinant lysophosphatidylcholine acyltransferase 3 (LPCAT3). ACSL4 is known to be positively correlated with susceptibility to ferroptosis, and the loss of LPCAT3 protects cells from damage caused by ferroptosis [37]. In turn, ferroptosis signals can be transmitted under lipid oxygenase (LOX) peroxide [110, 111]. Among different kinds of LOXs, arachidonate 15-lipoxygenase (ALOX15) is believed to play a core role in lipid peroxidation and ferroptosis [112]. Therefore, the combined action of ACSL4, LPCAT3, and ALOX15 can produce overwhelming lipid peroxidation in the cell, leading to the occurrence of cellular ferroptosis. In addition, phosphatidylethanolamine (PE), containing arachidonic acid (AA) and its derivative adrenal acid – a type of oxidized PL – has been shown to be the phospholipid most likely to induce ferroptosis [113].

#### The application of exosome-regulated ferroptosis in treating different diseases through the lipid metabolism pathway

Lipid metabolism plays an important role in tumorigenesis, invasion, metastasis, and many other tumour processes [114]. It has been shown that exosomes can regulate the function of target cells by secreting their contents into the tumour microenvironment and thus affect the occurrence and development of tumours [115, 116]. Additionally, the regulatory effect of exosomes on tumours is also associated with ferroptosis [117]. As a result, many studies have focused on ferroptosis and lipid metabolism to investigate the regulatory effects of exosomes in cancer cells. Using mass spectrometry of clinical samples from gastric cancer, Zhang and his team found that the levels of ALOX15 decreased, but the levels of ubiquitin-specific protease 7 (USP7) and heterogeneous nuclear ribonucleoprotein A1 (hnRNPA1) increased significantly in tumour tissue. By establishing relevant cell models, such as primary stromal cells and cancer cells, they found that cancer-associated fibroblast (CAF)-derived exosomes can secrete miR-552, a potential inhibitor of ALOX15, and hnRNPA1 as well as USP7 can increase the secretion and expression of miR-552 in exosomes. Cisplatin and paclitaxel promote the secretion of miR-552 from CAFs through the USP7/hnRNPA1 axis. This leads to the inhibition of ALOX15 and a reduction in lipid ROS accumulation in cancer cells, ultimately leading to a reduction in chemotherapy sensitivity [118]. Similarly, in 2021, the same team discovered that exosomal lncFERO (exo-lncFERO) secreted by gastric cancer cells could regulate the tumour-inducing properties of gastric cancer stem cells (GCSCs) by regulating ferroptosis of GCSCs through lipid ROS [26]. Recent studies have found that lipid metabolism pathways in GCSCs are significantly upregulated compared to those in differentiated GC cells, and stearoyl-coa-desaturase (SCD1), an enzyme that regulates the production of tumour lipid ROS, has the highest expression level [27, 119, 120]. Based on this, in vitro and in vivo experimental data provided by Zhang and his team demonstrated that exo-lncFERO can interact with SCD1 in GCSCs and recruit hnRNPA1 to promote the expression of SCD1. This leads to the dysregulation of PUFA levels in GCSCs and subsequent inhibition of ferroptosis. In addition, hnRNPA1 also enhanced the secretion of exo-lncFERO by GC cells, indicating that the exo-lncFERO/hnRNPA1/SCD1 axis can control the tumorigenic properties and resistance to chemotherapy of GCSCs [26]. Previous evidence suggests that obesity is strongly associated with cancer progression and poor prognosis [121, 122]. Zhang et al. found that microsomal triglyceride transfer protein (MTTP) expression was increased in plasma exosomes of colorectal cancer (CRC) patients with a high body fat rate through clinical studies. This increased expression reversed the occurrence of ferroptosis in CRC cells and made them resistant to oxaliplatin chemotherapy. Using transfection of KD-MTTP lentivirus into the abdominal fat of obese mice, a mechanistic study in colorectal cancer organoids found that adipocyte-derived exosomes could reduce the level of PUFA by upregulating GPX4 and xCT through the MTTP/PRAP1/ZEB1 axis, thereby inhibiting the production of lipid ROS and the incidence of ferroptosis [123]. In addition, the anti-ferroptosis properties of lung adenocarcinoma (LUAD) are also regulated by exosomes on lipid metabolism. By analysing plasma and tissue samples from healthy individuals and LUAD patients, Wang et al. found that plasma-derived exosomes from LUAD patients reduced lipid peroxidation. Further analysis of the LUAD mouse model showed that this phenomenon may be attributed to the increased expression of circRNA_101093 (cir93) in LUAD cells by exosomes. Cir93 can interact with fatty acid binding protein 3, thereby enhancing the transport of AA and its reaction with taurine and reducing the overall level of AA in the cell. Additionally, NAT, the reaction product of taurine and AA, can also inhibit the infiltration of AA into the plasma membrane by inhibiting ACSL4, LPCAT3, etc., and finally increase the ferroptosis resistance of LUAD [28]. In addition to its role in cancer, exosome regulation of ferroptosis via the lipid metabolic pathway is equally important in AKI. Sun et al. established a mouse model of AKI induced by IRI. It was demonstrated that human urine-derived stem cell-derived exosomes (USC-Exos) enriched with the lncRNA taurine-upregulated gene 1 (TUG1) effectively alleviated cell damage and ferroptosis. The potential mechanism may be related to the interaction between RNA binding protein serine/arginine splicing factor 1 (SRSF1) and ACSL4 mRNA stability [36].

## Conclusions

Research has indicated that the modulation of ferroptosis could offer a novel approach for the treatment of specific ailments. As regenerative medicine continues to advance, exosome-regulated ferroptosis in disease treatment has garnered increasing scholarly interest. Simultaneously, we have observed that the application of exosome-regulated ferroptosis is less relevant in the context of inflammatory diseases and other fields. Inflammation, as a pivotal pathological process underlying numerous diseases, typically arises from detrimental stimuli such as infection and tissue damage, thereby initiating a complex biological response [38]. Intriguingly, ferroptosis exhibits a close association with inflammation. Studies have demonstrated that intracellular iron overload can promote M1 macrophage polarization and amplify inflammatory responses [124]. Furthermore, during the course of inflammation, disruption in iron homeostasis occurs alongside excessive tissue and cellular damage [125]. In brief, the interaction between ferroptosis and inflammation in inflammatory diseases has the potential to exacerbate the progression of the disease. Consequently, employing a combination of anti-ferroptosis and anti-inflammatory therapies may disrupt this detrimental cycle and potentially achieve synergistic effects (1 + 1 > 2). Currently available evidence suggests that exosomes can alleviate inflammatory bowel disease through inhibition of ferroptosis; however, further investigations are warranted to elucidate the precise mechanisms involved [126].

Furthermore, ferroptotic cells can elicit proinflammatory damage-associated molecular patterns (DAMPs) and trigger immune responses [127]. The interplay between ferroptosis and immune cells has garnered significant attention, particularly in the context of antitumour effects. Ferroptosis in tumour cells leads to the release of various immune-stimulatory signals, which can facilitate dendritic cell maturation, enhance macrophage phagocytosis of ferroptotic cells, and further augment CD8^+^ Tcells infiltration into tumours [128]. Moreover, exosome delivery carrier characteristics offer additional possibilities for combating tumour drug resistance by leveraging ferroptosis. Consequently, the combination of exosome-mediated ferroptosis and tumour immune activation has become a new antitumour immunotherapy strategy. Cai et al. developed a nanoreactor Cu a ZIF-8 coated surface layer loaded with the ferroptosis agonist erastin to activate cancer cell ferroptosis while influencing TAM phenotypic approach. This approach also increased IFNγ secretion by CD8^+^T cells and further promoted erastin-induced ferroptosis mediated by the nanoreactor [129]. Additionally, Wang et al. harnessed exosome properties along with ferroptosis to construct mixed nanoparticles comprising a biocompatible oleic acid-Fe_3_O_4_ core attached with oxaliplatin polymers and Prominin2 siRNA. These nanoparticles inhibited iron efflux from tumour cells while enhancing their susceptibility to undergo ferroptosis and activating their immunogenicity against metastasis [130].

**Fig. 1:**
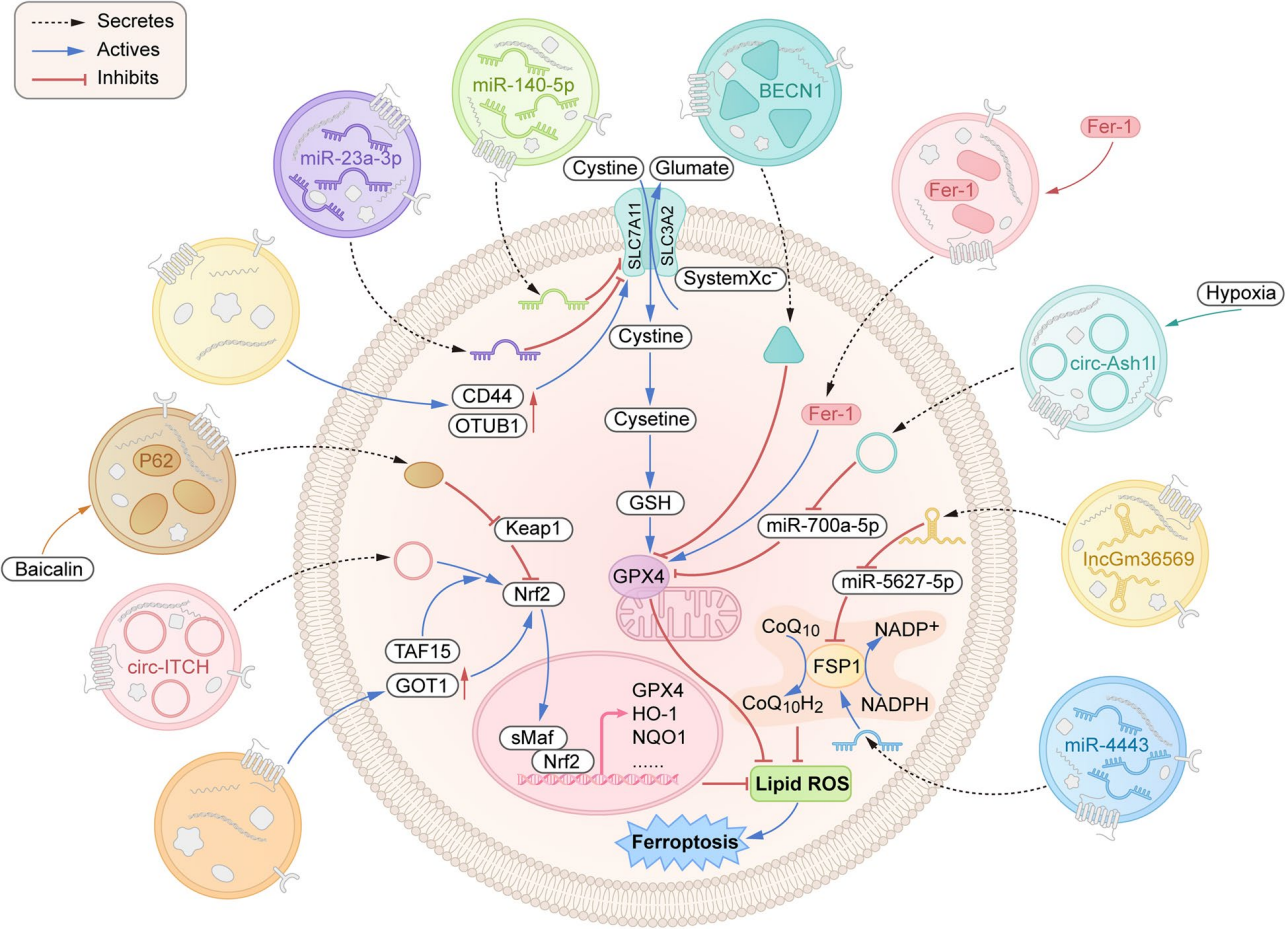
Mechanisms of exosome-regulated ferroptosis through “defence pathways”. The system Xc-/GSH/GPX4 and NAD(P)H/FSP1/CoQ10 axes serve as two “defence pathways” to protect cells from ferroptosis. In addition, the activation of the Nrf2 antioxidant pathway cooperated with GPX4 to inhibit lipid peroxidation and ferroptosis. Exosomes derived from different cells can directly or indirectly regulate the above pathways through their functional components to regulate the occurrence and development of ferroptosis in cells. This diagram illustrates the pathway by which exosomes regulate ferroptosis by inhibiting or promoting the key targets SLC7A11, GPX4, FSP1, and Nrf2. SLC7A11: solute carrier family 7 member 11; GPX4: glutathione peroxidase 4; FSP1: ferroptosis suppressor protein 1; Nrf2: nuclear factor E2-related factor 2

**Fig. 2:**
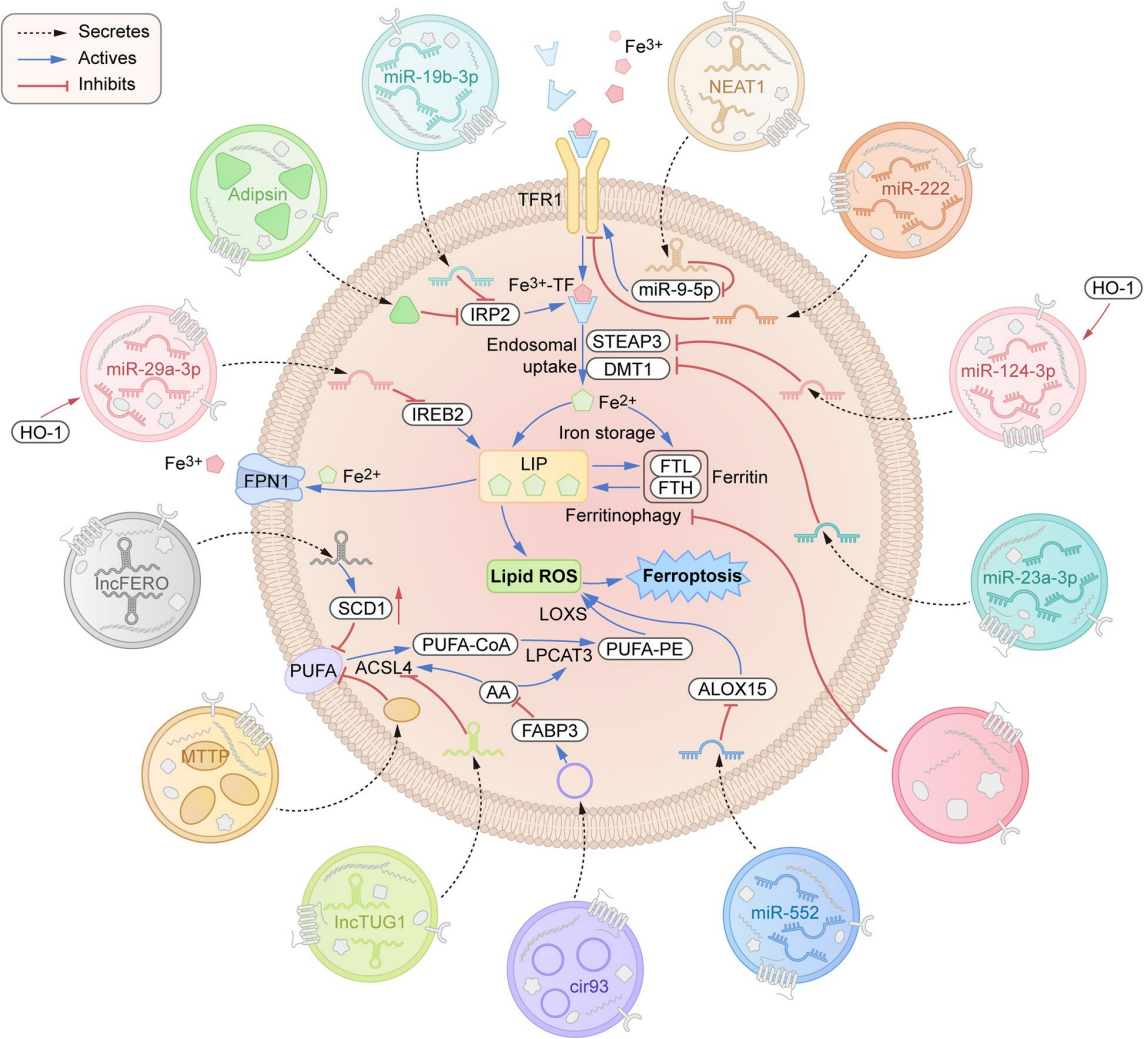
Mechanisms of exosome-regulated ferroptosis through the “offensive pathway”. Iron metabolism and lipid metabolism pathways serve as two “offensive pathways” that can promote ferroptosis by promoting the accumulation of intracellular iron and lipid peroxidation. Exosomes derived from different cells can directly or indirectly inhibit iron metabolism and lipid metabolism pathways to inhibit the occurrence of intracellular ferroptosis. The key targets of exosomes in regulating lipid metabolism pathways included TFR1, IRP2, IREB2, STEAP3, and DMT1. The key targets of exosomes in regulating lipid metabolism pathways included PUFA, ACSL4, and ALOX15. TFR1: transferrin receptor 1; IRP2: iron regulatory protein 2; IREB2: iron response element-binding protein 2; STEAP3: six-transmembrane epithelial antigen of prostate 3 metalloreductase; DMT1: divalent metal transporter 1; PUFA: polyunsaturated fatty acid; ACSL4: acyl-CoA synthetase long-chain family member 4; ALOX15: arachidonate 15-lipoxygenase

Overall, this review provides a comprehensive analysis, surpassing prior studies, to systematically elucidate that exosomes can regulate ferroptosis through four main pathways: system Xc-/GSH/GPX4, system NAD(P)H/FSP1/CoQ10, iron metabolism and lipid metabolism (Figs. 1 and  2). Based on the new insight that reasonable induction or inhibition of ferroptosis can regulate disease progression, we believe that ferroptosis may be the most promising candidate for exosome influence on the progression of different diseases. However, there remain several questions that necessitate attention prior to the broader implementation of exosome-regulated ferroptosis. Additional investigations are imperative to ascertain the safety, dosage response, and potential adverse effects of exosomes. However, the understanding of the upstream and downstream mechanisms involved in exosome-mediated ferroptosis, as well as its potential therapeutic applications, remains limited due to a lack of investigations. Additionally, the present study mostly focused on the regulation of nucleic acid molecules in exosomes, neglecting a comprehensive exploration of the impact of protein and lipid molecules on ferroptosis. Moreover, much of the current research is based on animal models and cell experiments, but there is a lack of connection between these basic studies and clinical practice.

Consequently, conducting further comprehensive investigations into the correlation between exosomes and ferroptosis, in conjunction with the management of inflammation, immunity, and other related aspects, will broaden the potential applications of exosome-mediated regulation of ferroptosis across various disease domains. Additionally, this will offer a more defined trajectory and a more robust theoretical foundation for the clinical exploration of exosome-mediated regulation of ferroptosis. Furthermore, a promising direction for future research is to investigate whether exosomes that regulate other emerging forms of cell death, such as cuproptosis, are also involved in disease progression [131]. In any case, the exploration of exosome-regulated ferroptosis may provide benefits in disease intervention and treatment.

## Data Availability

Not applicable.

## Abbreviations

ROS: reactive oxygen species
IRI: ischaemia–reperfusion injury
LIP: labile iron pool
MVBs: multivesicular bodies
miRNA: microRNA
lncRNA: long non-coding RNA
SLC3A2: Solute carrier family 3 member 2
SLC7A11: Solute carrier family 7 member 11
GSH: glutathione
NFE2L2: erythroid 2-like 2
BAP1: BRCA1-associated protein 1
GPX4: glutathione peroxidase 4
HFD: high-fat diet
ATM-Exo: adipose tissue macrophage-derived exosomes
CF-Exo: cardiac fibroblast derived exosomes
MSC-Exo: mesenchymal stem cell-derived exosomes
ALI: acute liver injury
CCL4: carbon tetrachloride
I/R: ischemia/reperfusion
BMMSC-EVs/Fer-1: Fer-1 loaded bone marrow mesenchymal stem cell-derived extracellular vesicles
ADSC: adipose-derived stem cell
HExo: hypoxia-pretreated adipose-derived stem cells exosomes
DFU: diabetic foot ulcer
circ-ITCH: circRNA-itchy E3 ubiquitin protein ligase
TAF15: TATA-Box binding protein associated factor 15
HSCs: hepatic stellate cells
HucMSC-Exo: human umbilical cord mesenchymal stem cell-derived exosomes
CCR2: C–C motif chemokine receptor-2
FSP1: ferroptosis inhibitor protein 1
NSCLC: non-small cell lung carcinoma
METTL3: methyltransferase-like 3
ASCI: acute spinal cord injury
Fe^3+^: extracellular iron
TF: transferrin
TFR1: transferrin receptor protein-1
Fe^2+^: ferrous iron
STEAP3: six-transmembrane epithelial antigen of prostate 3 metalloreductase
DMT1: divalent Metal Transporter 1
Zip8/14: zentz-iron regulatory protein family 8/14
FTL1: ferritin light peptide 1
FTH1: ferritin heavy peptide 1
FPN: ferroprotein
IREB2: iron response element binding protein 2
SAE: sepsis associated encephalopathy
NEAT1: nuclear-enriched transcript 1
GOT1: glutamic oxaloacetic transaminase 1
MI: myocardial infarction
IRP2: iron regulatory protein 2
HO-1/BMMSCs: modification of BMMSCs by heme oxygenase-1
SHP-HR: HR model of fatty liver cells
EC-Exo: endothelial cell-derived exosomes
KEGG: Kyoto Encyclopedia of Genes and Gnomes
PUFAs: polyunsaturated fatty acids
ACSL4: acyl-CoA synthase long chain family member 4
PLs: phospholipids
LPCAT3: Lysophosphatidylcholine Acyltransferase 3
LOXs: lipid oxygenase
ALOX15: arachidonate 15-lipoxygenase
PE: phosphatidylethanolamine
AA: arachidonic acid
USP7: ubiquitin-specific protease 7
hnRNPA1: heterogeneous nuclear ribonucleoprotein A1
CAFs: cancer-associated fibroblasts
exo-lncFERO: exosome lncFERO
GCSCs: gastric cancer stem cells
SCD1: stearoyl-Coa-desaturase
MTTP: microsomal triglyceride transfer protein
CRC: colorectal cancer
LUAD: lung adenocarcinoma
cir93: circRNA_101093
USC-Exo: human urine-derived stem cells-derived exosomes
TUG1: taurine-upregulated gene 1
1SRSF1: Serine/arginine splicing factor 1
